# Gonadotropin Glycoforms Circulating in Women Using Progestins of the Levonorgestrel Family for Contraception

**DOI:** 10.1210/jendso/bvaa128

**Published:** 2020-08-28

**Authors:** Karin Eriksson, Leif Wide

**Affiliations:** Department of Clinical Chemistry, University Hospital, Uppsala, Sweden

**Keywords:** N-glycosylation, sulfonation, sialylation, levonorgestrel, progestin, contraception

## Abstract

**Context:**

The progestins of the levonorgestrel family are 13-ethylgonane progestins, commonly used for contraception in women. One contraceptive effect of these progestins is inhibition of ovulation, which may be a result of changes in gonadotropin glycosylation patterns. Gonadotropin glycoforms differ in number of glycans and bioactivity: more bioactive low-N-glycosylated glycoforms, diglycosylated luteinizing hormone (LHdi) and triglycosylated follicle-stimulating hormone (FSHtri), and less bioactive fully N-glycosylated glycoforms, LHtri and FSHtetra.

**Objective:**

Characterize the glycosylation patterns on the circulating gonadotropin glycoforms in women using 13-ethylgonane progestins for contraception.

**Design, Subjects, Main Outcome Measures:**

Serum samples, collected from 92 healthy women using 13-ethylgonane progestins for contraception, were included. Forty women used progestin-only continuously and 52 used progestins combined with ethinylestradiol (EE) for 3 weeks followed by a hormone-free week. Concentration, sulfonation, and sialylation of each glycoform were determined and compared with follicular phase values of normal menstrual cycles.

**Results:**

The progestin-only group had significantly increased serum levels, decreased sulfonation, and increased sialylation of LHdi. The LHdi/FSHtri ratio was increased. The progestin+EE group had significantly decreased gonadotropin glycoform concentrations and decreased sialylation of FSHtri. The progestin+EE effect on sialylation of FSHtri occurred later during the treatment cycle in contrast to the effect on FSHtri concentration.

**Conclusions:**

The 2 different progestin treatments induced different effects on the glycan synthesis and concentrations of more bioactive low-glycosylated gonadotropins. Progestin-only treatment increased sialylation and decreased sulfonation of LHdi molecules, contributing to sustained higher levels of bioactive LHdi molecules. Progestin+EE treatment decreased sialylation of FSHtri, contributing to a shorter half-life and decreased levels of bioactive FSHtri.

The levonorgestrel family of synthetic progestins are characterized by having an ethyl group at gonane carbon 13 and an ethinyl group at carbon 17 [1, 2]. The term gonane is used for the basic 17-carbon steroid nucleus, the cyclopentanoperhydrophenanthrene, and the term progestin for a synthetic progestogen [3, 4]. These 13-ethylgonane progestins are commonly in use for contraception in women. In fact, among 742 female medical students in Uppsala (2000-2011), 46% used hormonal contraceptives, and among them the majority, or 62% (196 women), used 13-ethylgonane progestins of the levonorgestrel family: levonorgestrel (LNG), desogestrel (DSG), or etonogestrel (ENG). The treatments were either continuous with progestin-only or combined with ethinylestradiol (EE) (progestin+EE), administered for 21 days followed by a 7-day hormone-free interval.

One of the contraceptive effects of the 13-ethylgonane progestins is an interference with the hypothalamo-pituitary-ovarian axis, leading to suppression of follicular activity and anovulation [5]. A sophisticated interplay between the ovary, the pituitary gland and the hypothalamus regulate the events leading to follicular development, ovulation, and corpus luteum formation during the normal ovulatory ovarian cycle [6]. The ovarian steroids regulate the pulsatile secretion of the gonadotropins and modify the biological properties of the gonadotropins by changing their degrees of N-glycosylation, sialylation, and sulfonation [7, 8].

Follicle-stimulating hormone (FSH) exists in human pituitary extracts as different glycoforms. All the FSH glycoforms are N-glycosylated with 2 glycans on the alpha subunit, while their beta subunits are N-glycosylated with 0, 1, or 2 glycans [9]. We have found that all 3 pituitary glycoprotein hormones, FSH, luteinizing hormone (LH), and thyrotropin (thyroid-stimulating hormone; TSH) circulate in blood as low-N-glycosylated and fully N-glycosylated forms differing in total number of glycans: FSHtri and FSHtetra; LHdi and LHtri; and TSHdi and TSHtri, respectively [10]. The low N-glycosylated forms of both FSH and LH play major roles in the natural ovarian stimulation [11]. The degrees of sialylation and sulfonation of the N-glycans differ with the phases of the menstrual cycle, after menopause, and in women with polycystic ovary syndrome [11, 12]. The low-N-glycosylated forms of FSH, LH, and TSH are less sialylated and such molecules exhibit a higher bioactivity at the target cells compared with the more sialylated fully N-glycosylated forms [13-24].

We hypothesized that abnormal glycosylation patterns on the circulating gonadotropins may contribute to suppression of follicular activity and anovulation during treatment with progestin-only or progestin+EE in young women. To address this question we have analyzed the gonadotropin glycoforms in one serum sample from each of 92 female medical students who used 13-gonane progestins for contraception. The serum concentrations and degrees of sialylation and sulfonation of low- and fully N-glycosylated glycoforms of FSH and LH were determined as previously described [10]. Follicular phase values of 26 young women with normal menstrual cycles were used for statistical comparisons.

## 1. Participants and Methods

### Participants

As part of the training in clinical chemistry for medical students at Uppsala University Hospital, blood samples were taken on one occasion from each of about 120 students each year during the period 2000 to 2011. The blood samples were taken on an ambulatory basis between 8 am and 9 am after an overnight fast and were evenly distributed from September to May. Almost all the students agreed to donate one serum sample to a research project and signed a detailed health declaration. The research project aimed to explore the physiological and clinical significance of the glycobiology of FSH, LH, and TSH. The donation of a serum sample was accompanied with informed consent according to the Declaration of Helsinki of Ethical Principles for Medical Research and with approval of the Ethic Committee of the Medical Faculty of Uppsala University.

Ninety-two female students using 13-ethylgonane progestins for contraception, median age 24 years (range, 21-41 years), were considered suitable for the present study. Forty women used 13-ethylgonane progestins-only, and 52 used 13-ethylgonane progestins in combination with EE (Table 1). They were all apparently healthy, had no history of endocrine disease and used the progestins exclusively for contraception. They had all supplied us with detailed information about their use of the progestins. They had serum FSH and LH concentrations of >0.10 U/L when initially measured, which was the lower limit for reliable measurements by the electrophoresis.

**Table 1.** 13-Ethylgonane Progestins, Modes of Administration, and Number of Women

Group	Progestin	Mode of administration	FSH	LH
			n	n
Progestin-only, treated continuously	Levonorgestrel	Intrauterine device	5	5
	Desogestrel	Oral, daily tablet	20	19
	Etonogestrel	Subdermal implant	15	14
		Total	40	38
Progestins combined with ethinylestradiol during 28-day cycles; including 3 weeks of treatment and 1 hormone-free week	Levonorgestrel	Oral, monophase or triphase	38	36
	Desogestrel	Oral, monophase	10	9
	Etonogestrel	Vaginal ring	4	4
		Total	52	49

Both the progestin-only and the progestin+EE group included 3 subgroups (Table 1). Statistical comparisons of results for the 3 different subgroups were made for all the different variables analyzed in this study. No significant deviation was detected for any of the subgroups, which rendered them all suitable to be pooled into either the progestin-only or the progestin+EE group.

A reference group of follicular phase values from normal menstrual cycles comprised the results of analyses of one serum sample from each of 26 medical students taken on days 1 to 11 of the cycle. This follicular phase group was derived from a previously reported study on 78 healthy medical students having normal menstrual cycles [11]. The follicular phase serum samples were obtained in parallel with those of the present study and the analytical methods used were identical.

During the period autumn 2005-2011 the body mass index (BMI) was recorded for the 2 progestin groups and the follicular phase group of women. The mean value for 29 women of the progestin-only group was 21.6 (range, 19.3-28.7); for 26 women of the progestin+EE group 20.8 (18.3-25.7); and for 9 women of the follicular phase group 21.6 (19.5-28.7). During the same period the free testosterone mean serum level and range for 20 women of the progestin-only group was 1.26 nmol/L (range, 0.53-1.92), and for 10 women of the follicular phase group 1.23 nmol/L (0.75-2.1).

### Immunoassay of serum FSH and LH

The concentrations of FSH and LH in serum samples and in separated fractions after electrophoreses were measured using time-resolved sandwich fluoroimmunoassays (Delfia, PerkinElmer-Wallac Oy, Turku, Finland) [25, 26]. The methods permitted measurements of the hormones directly in the 0.075 M veronal (Sigma-Aldrich Chemie Gmbh, Germany) buffer at pH 8.7 eluted from electrophoreses. All sera were initially tested to identify individuals with the common variant form of LH [27]. Gonadotrophin values were expressed in IU/L using the International Standards for pituitary LH (80/552) and FSH (94/632) as reference standards. The detection limits were less than 0.02 IU/L serum and the interassay coefficient of variation (CV) was less than 3% for both hormones. The detection limit of the 2 hormones in fractions from electrophoresis was about 100 attogram.

### Frequency of glycoforms of FSH and LH and median numbers of anionic monosaccharide residues per glycoform molecule

All serum samples were analyzed with an electrophoresis technique using a 0.10% agarose suspension in veronal buffer at pH 8.7. The motilities were expressed in relation to that of endogenous human serum albumin as Albumin Mobility Units. The area of eluted gonadotropin was resolved into peaks at the positions for different numbers of anionic monosaccharide (AMS) residues per molecule. The frequencies of the 2 glycoforms of LH and of FSH in serum samples and the median numbers of AMS residues per glycoform molecule were calculated from the distribution by electrophoresis using the FSH and LH algorithms as described [10].

### Neuraminidase treatment

The terminal sialic acid (SA) residues were removed from the LH and FSH molecules, both in serum samples and in separated fractions after electrophoresis, by neuraminidase treatment during 24 hours at 37 °C, leaving the sulfonated N-acetylgalactosamine (SU) as the only AMS remaining on the molecules, as described [10].

### Determination of SU and SA on glycoforms

The number of SU residues and the percentage of SU out of the AMS were determined for each glycoform. The ratio of percentage SU out of total AMS per molecule on low versuss fully N-glycosylated glycoforms was 1.186 for LH and 1.349 for FSH of the progestin-treated women.

These factors were used to calculate the number of SU and SA per glycoform molecule in the serum samples, as described [10].

### Statistical analyses

Mean values are presented with CV in percent. Statistical comparisons were made by using a nonparametric Mann-Whitney test. The time-related dynamic change, during the progestin+EE 28-day treatment cycles, for FSHtri concentration versus degree of sialylation, are presented as 3-day moving mean values ± standard error of the mean (SEM). A difference with a *P* value < 0.01 was considered significant.

## 2. Results

### Effects of progestin-only and progestin+EE on gonadotropin glycoform concentration and degrees of sialylation and sulfonation

The mean values with CV percentage for the follicular phase group, the progestin-only group and the progestin+EE group together with *P* values of statistical comparisons with the follicular phase group for 8 variables related to glycoform serum concentration and 8 variables related to glycan modification are presented in Table 2.

**Table 2.** FSH and LH Glycoform Serum Concentration, N-glycosylation and Glycan Modification in Women Treated With 13-Ethylgonane Progestin-only or With Progestin+EE for 21 Days followed by a Hormone-Free Interval (HFI) of 7 days. Follicular Phase Values of Women With Normal Menstrual Cycles Used for Statistical Comparisons Using Nonparametric Mann-Whitney Test

Number of women FSH/LH	Group A, follicular phase, day 1-11 of the menstrual cycle 26/26 (mean; CV%)	Group B, progestin-only, continuous treatment 40/38 (mean; CV%)	Group C, progestin+EE, 21 days plus 7 days HFI 52/49 (mean; CV%)
***Glycoform concentration***			
LHdi, IU/L	1.40; 36.0	2.39; 61.5^*a*^	1.34; 97.2^ns^
LHtri, IU/L	2.53; 35.6	3.43; 50.2^ns^	1.55; 75.4^*c*^
N-glycosylation of LH, %^*d*^	63.9; 15.5	59.8; 15.9^ns^	56.3; 24.3^ns^
FSHtri, IU/L	2.25; 32.6	1.79; 44.4^ns^	1.56; 72.0^*b*^
FSHtetra, IU/L	3.13; 25.0	3.48; 37.9^ns^	2.00; 60.1^*c*^
N-glycosylation of FSH, %^*d*^	58.5; 10.5	65.2; 18.3^*a*^	56.5; 22.1^ns^
Ratio LHdi/FSHtri	0.67; 43.2	1.44; 73.0^*c*^	0.94; 65.7^ns^
Ratio LHtri/FSHtetra	0.83; 34.0	0.99; 40.4^ns^	0.88; 54.4^ns^
***Glycan modification***			
LH			
SA per LHdi molecule	1.31; 9.22	1.51; 11.9^*c*^	1.32; 18.4^ns^
SU per LHdi molecule	1.13; 10.4	0.94; 18.0^*c*^	1.15; 19.0^ns^
SA per LHtri molecule	2.27; 6.47	2.47; 9.28^*c*^	2.22; 13.8^ns^
SU per LHtri molecule	1.32; 10.2	1.18; 17.8^*b*^	1.44; 18.9^ns^
FSH			
SA per FSHtri molecule	5.58; 1.46	5.59; 3.14^ns^	5.43; 3.8^*b*^
SU per FSHtri molecule	0.31; 25.3	0.25; 73.2^ns^	0.43; 50.0^*a*^
SA per FSHtetra molecule	7.05; 0.90	7.14; 2.29^*b*^	6.99; 2.83 ^ns^
SU per FSHtetra molecule	0.28; 25.4	0.23; 72.9 ^ns^	0.40; 50.0^*a*^

Abbreviations: CV, coefficient of variation; EE, ethinylestradiol; FSH, follicle-stimulating hormone; LH, luteinizing hormone; ns, not significant (*P* > 0.01); SA, sialic acid; sulfonated N-acetylgalactosamine.

^*a*^ *P* < 0.01; ^*b*^*P* < 0.001; ^*c*^*P* < 0.0001.

^*d*^ Degree of N-glycosylation expressed as percentage of fully N-glycosylated glycoforms, ie, FSHtetra or LHtri.

#### Progestin-only group versus follicular phase group.

 The LHdi concentration and the ratio of low-glycosylated glycoforms (LHdi/FSHtri) were significantly higher in the progestin-only group compared with the follicular phase group. The degree of N-glycosylation of FSH was higher. Both LH glycoforms were more sialylated and less sulfonated. The FSHtetra molecules were more sialylated.

#### Progestin+EE group versus follicular phase group.

 The serum levels of LHtri, FSHtri, and FSHtetra were significantly lower in the progestin+EE group compared with the follicular phase group. The degree of sialylation was significantly lower for FSHtri and that of sulfonation higher on both FSH glycoforms.

#### Progestin+EE group versus progestin-only group.

 The progestin+EE group had significantly (*P* < 0.0001) lower serum concentrations of both LH and FSH glycoforms and all 4 glycoforms were significantly (*P* < 0.001) less sialylated and more sulfonated compared with the progestin-only group.

### Dynamic changes of FSHtri serum concentration and degree of sialylation during progestin+EE treatment cycles

**Figure 1.**
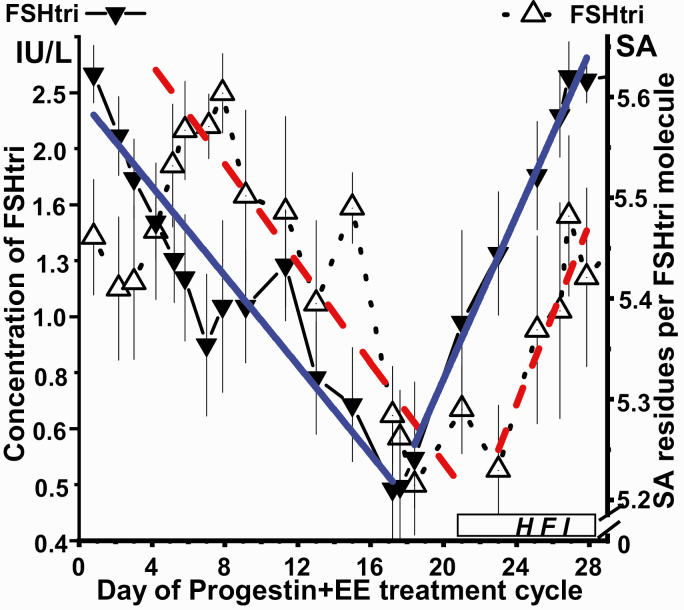
Dynamic changes, using 3-day moving mean values ± SEM, of serum concentration of FSHtri, *filled symbols,* and number of sialic acid (SA) residues, *open symbols*, per FSHtri molecule during 28-day progestin+EE treatment cycles. The treatments cycles included administration of progestin+EE for 21 days, followed by a 7-day hormone-free interval (HFI). The blue filled straight lines indicate, during the 2 periods of the treatment cycle, the linear relationship between time and concentration, and the red hatched straight lines the corresponding relationship for number of sialic acid residues per molecule.

The time-related dynamic change during the 28-day progestin+EE treatment cycle of FSHtri concentration compared with that of degree of sialylation is presented in Fig. 1, using 3-day moving mean values ± SEM. The concentration of FSHtri responded to the hormone treatment with a gradual decrease from day one, to a minimum level during the third week of treatment and then a rapid rise during the hormone-free interval. The decrease and increase of the degree of sialylation of FSHtri occurred with a delay of 4 to 6 days when compared with the effects on FSHtri serum concentration.

## 3. Discussion

We report in this study that the glycosylation pattern on gonadotropin glycoform molecules in serum samples of women using, for contraception, progestin-only or progestin+EE is different from that during the follicular phase of normal menstrual cycles. The glycosylation patterns of the progestin-only and the progestin+EE treatment groups differed considerably, suggesting different modes of interfering with the normal follicular maturation and ovulation.

Women taking progestin-only had raised serum levels of the more biologically active low-N-glycosylated glycoform, LHdi, and these molecules were less sulfonated and more sialylated than during the follicular phase of the normal cycle. LH molecules with 2 or more SU residues per molecule are quickly removed from the circulation by an SO3-N-acetylgalactoseamine receptor in the liver [28, 29]. A decreased degree of sulfonation of the LH molecules will contribute to increased serum levels. SA residues prolong the half-life of the LH molecules in the circulation, and increased sialylation contributes to the raised LHdi serum concentration compared with the follicular phase of the cycle [30].

Women taking progestin-only had significantly increased ratio of the low-N-glycosylated forms, LHdi/FSHtri. It is well established that the ratio of LH/FSH concentration is raised in women with polycystic ovary syndrome, and we have observed that in these women it is, in particular, the ratio of the more biologically active low-N-glycosylated forms, LHdi/FSHtri, which is increased (unpublished observation). The similarity in this respect is noteworthy.

Treatment with progestin+EE included a hormone administration during 21 days and then a 7-day hormone-free interval. The progestin+EE treatment decreased the mean levels of FSHtri, FSHtetra, and LHtri compared with the mean values of the follicular phase and decreased the sialylation of FSHtri. The decreased sialylation of FSH by the EE treatment is in agreement with previously observed effects of 17β-estradiol in postmenopausal women [31]. The low degree of sialylation of FSHtri molecules during the 28-day treatment cycle is expected to contribute to the low mean serum level of the FSHtri glycoform during this treatment.

The dynamic changes in the concentration of FSHtri and in the degree of sialylation of this molecule during the 28-day progestin+EE treatment cycle are illustrated in Fig. 1. The results revealed a phase shift in time during the 28-day treatment cycle for the FSHtri concentration compared with its degree of sialylation. The concentration of FSHtri responded to the hormone treatment with a gradual decrease from day one, to a minimum level during the third week of treatment, followed by a rapid rise during the hormone-free interval. The dynamic changes of the degree of FSHtri sialylation, measured as number of SA residues per molecule, occurred 4 to 6 days later than that observed for the FSHtri concentration, both during the hormone treatment period and during the hormone-free interval.

This observation on the degree of sialylation of FSHtri, with a delay of 4 to 6 days, excludes that the variation in sialylation caused the dynamic changes in FSHtri concentration during the 28-day treatment cycle. It seems more likely that the dynamic effects on the FSHtri concentration during this treatment was due to a direct effect on the hypothalamus-pituitary axis, decreasing the synthesis and secretion of FSH.

Enzymes regulate the terminal sialylation on the glycans. The 4 to 6 days later responses of the glycan terminal sialylation during the progestin+EE treatment cycles indicate the existence of some control mechanism in the Golgi. This mechanism may have been developed to protect against effects of occasional variations in hormone secretions.

In conclusion, the different effects on low-glycosylated gonadotropin glycoforms by progestin-only versus progestin+EE treatment suggests different modes of action in inhibiting ovulation. The progestin-only treatment induced increased sialylation and decreased sulfonation of LHdi molecules, contributing to sustained higher serum concentrations of the more biologically active LHdi molecules. The progestin+EE treatment resulted in decreased FSHtri and FSHtetra serum levels and decreased sialylation of FSHtri. The progestin+EE administration caused a prompt response of FSHtri serum concentration and a close to linear decrease up to the hormone-free period. The response to the hormone-free period was again prompt and followed by a close to linear increase. We suggest that the progestin+EE has a direct effect on the hypothalamus-pituitary axis leading to a decreased secretion of the more biologically active FSHtri molecules. The effects of progestin+EE on the sialylation of FSHtri occurred 4 to 6 days later in contrast to that on the FSHtri concentration. This excludes a possible causal relationship between the dynamic changes of sialic acid content and those of FSHtri serum concentration during the 28-day treatment cycle. The average degree of sialylation of FSHtri during the progestin+EE 28-day treatment cycle was low. This low degree of sialylation of FSHtri leads to a shorter circulatory half-life, which contributes to the decreased FSHtri serum levels.

## Data Availability

All data generated or analyzed during this study are included in this published article or in the data repositories listed in References.

## Abbreviations

AMS: anionic monosaccharide
CV: coefficient of variation
DSG: desogestrel
EE: ethinylestradiol
ENG: etonogestrel
FSH: follicle-stimulating hormone
FSHtri/tetra: tri/tetraglycosylated follicle-stimulating hormone
LH: luteinizing hormone
LHdi/tri: di/triglycosylated luteinizing hormone
LNG: levonorgestrel
SA: sialic acid
SU: sulfonated N-acetylgalactosamine
TSH: thyrotropin (thyroid-stimulating hormone)

## Additional Information

***Disclosure Summary:*** The authors have nothing to disclose.
